# Intestinal Obstruction Due to an Anomalous Congenital Band

**DOI:** 10.4103/1319-3767.37806

**Published:** 2008-01

**Authors:** Cyrochristos Dimitrios, Alexiou A. George, Ziogas Dimosthenis, Xiropotamos Nikolaos

**Affiliations:** Department of Surgery, University Hospital of Ioannina, Greece

**Keywords:** Congenital band, intestinal obstruction, remnant

## Abstract

We report a case of a 20-year-old male who presented with symptoms and signs of intestinal obstruction. The patient reported no previous history of abdominal surgery or trauma while clinical and radiographic examinations were not diagnostic. An open laparotomy was subsequently performed and the intraoperative findings were consistent with a congenital band extending from the antimesenteric wall of the jejunum to the root of mesentery. The band was ligated and divided with an uneventful postoperative course. Congenital bands are extremely rare. Their exact incidence is still unknown and usually observed in childhood. This case, therefore, represents an unusual surgical problem in an older individual in which the diagnosis was clinically unexpected.

Small bowel obstruction is the most common surgical disorder of the small intestine. Adhesions are by far the most frequent causes followed by hernias, tumors, intussusception, foreign bodies, gallstones, and inflammatory bowel disease.

Obstruction by a congenital band is extremely rare and usually observed in childhood.

This report presents a 20-year-old male with symptoms of intestinal obstruction, subsequently treated by ligation and division of a congenital band. This is the first report of a band running from the root of the mesentery to the jejunum.

## CASE REPORT

A 20-year-old male presented to the Emergency Department complaining of epigastric pain of 6-h duration. He also reported an episode of vomiting. There was no history of abdominal surgery or trauma. Abdominal palpation revealed moderate tenderness over the epigastric area without rebound tenderness or a palpable mass. On auscultation, there were active bowel sounds. Rectal examination was normal. At the time of arrival, he had a temperature of 37.0°C, a blood pressure of 130/80 mm Hg, and a pulse rate of 85 beats/min. Laboratory data showed an elevated lactate dehydrogenase of 241 IU/l and creatinophosphokinase of 507 IU/l, but no leukocytosis.

**Figure 1:**
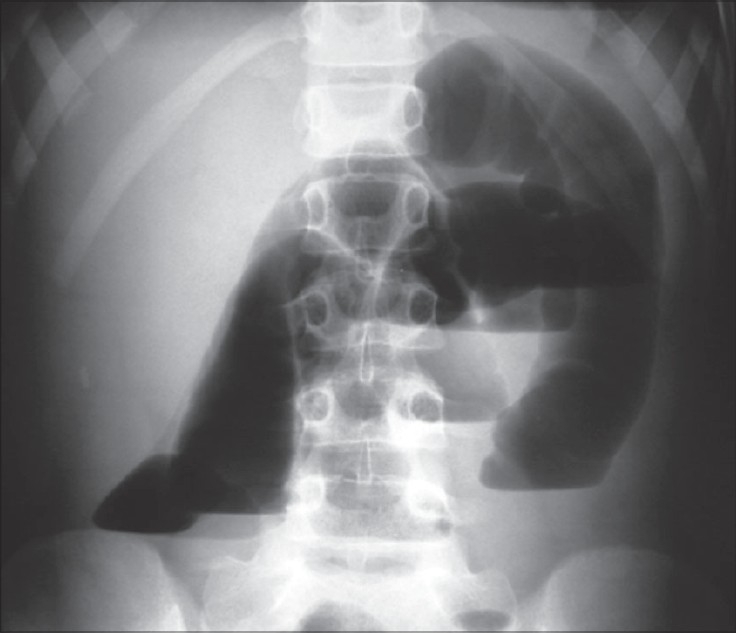
X-ray showing intestinal loops with air-fluid levels

**Figure 2:**
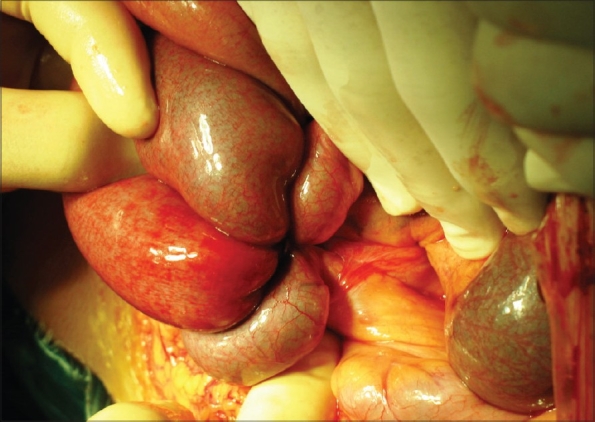
Findings at laparotomy showing the anomalous congenital band with no signs of ischemic bowel

Abdominal plain X-ray showed intestinal loops with air-fluid levels on the left side of the abdomen, which remained unaltered in a subsequent X-ray after 2 h [Figure 1]. Due to the patient's clinical deterioration, an exploratory laparotomy was performed and revealed a congenital vascular band that formed a closed loop through which a part of small bowel was entrapped. The band was vascularized by one of the branches of the superior mesenteric artery, and the start of the band was located at the end of the jejunum, and the termination was at the root of the mesentery at the level of the second lumbar vertebrae; no sign of ischemic bowel was noted [Figure 2]. The band was ligated and divided. Histopathological examination revealed a fibrotic band containing blood vessels. Postoperative outcome was uneventful and the patient was discharged on the fourth postoperative day.

## DISCUSSION

Congenital bands are a rare cause of intestinal obstruction in infancy and childhood. Their occurrence in adults is an extremely rare condition.[1,2] Obstruction is caused by entrapment of the intestine between the band and mesentery or by compression of the bowel. Akgur *et al.* have recently reported in a series of eight patients that bands principally were located between ascending colon and terminal ileum followed by ligament of Treitz and terminal ileum; between the right lobe of liver and terminal ileum; and between the right lobe of liver and ascending colon.[1] Lin *et al.* have reported a band extending from the iliac fossa to the sigmoid mesocolon,[3] while Itagaki *et al.* reported the presence of a jejuno-jejuno congenital band.[4] As far as we know, there are no reports of a band running from the root of mesentery to the jejunum. In addition, its location excluded known embryogenic remnants such as mesourachus or vitelline arteries, veins or omphalomesenteric ducts.[5,6] In all the reported cases, the band was well vascularized as was the case in the present study.

Patients usually present with symptoms of intestinal obstruction, and despite the availability and wide use of modern imaging techniques, preoperative diagnosis is very difficult to establish. Plain films are nonspecific. Ultrasound scan might provide details of localized distended intestinal loops or indirect signs of peritonitis, but it is not specific while barium-contrast gastrointestinal series may provide clues to narrow the differential diagnosis. In the present case, plain abdominal X-ray revealed air-fluid levels located on the left side of the abdomen, which remained unaltered in a subsequent image after 2 hours.

Concerning the management of a congenital band, surgical treatment is the cornerstone. Traditionally, laparotomy is indicated, whereas with the advent of minimally invasive surgery, laparoscopy has been proposed as an alternative. Wu *et al*. have recently reported that laparoscopy may be safe and feasible in the diagnosis and treatment of a congenital band.[7]

In conclusion, the possibility of a congenital band must be included in the differential diagnosis of young patients with symptoms and signs of bowel obstruction and no history of abdominal surgery, trauma or clinical hernia, although this entity is very uncommon. This clinical situation requires early surgical intervention that will be diagnostic and therapeutic.
